# Trans Health is Public Health: The Prevalence of HIV Among Trans and Gender Expansive People in Kazakhstan

**DOI:** 10.21203/rs.3.rs-5124958/v1

**Published:** 2024-09-24

**Authors:** Kelsey G. Reeder, Yong Gun Lee, Jimin Sung, Vitaliy Vinogradov, Gulnara Zhakupova, Gaukhar Mergenova, Alissa Davis, Emily Allen Paine, Sholpan Primbetova, Assel Terlikbayeva, Sultana Kali, Timothy Hunt, Elwin Wu

**Affiliations:** Social Intervention Group, Columbia University School of Social Work, New York, NY, USA; Columbia University Global Health Research Center of Central Asia, Almaty, Kazakhstan; Department of Social Work and Social Administration, The University of Hong Kong, Pokfulam, Hong Kong; Social Intervention Group, Columbia University School of Social Work, New York, NY, USA; Columbia University Global Health Research Center of Central Asia, Almaty, Kazakhstan; Columbia University Global Health Research Center of Central Asia, Almaty, Kazakhstan; Columbia University Global Health Research Center of Central Asia, Almaty, Kazakhstan; Columbia University Global Health Research Center of Central Asia, Almaty, Kazakhstan; Social Intervention Group, Columbia University School of Social Work, New York, NY, USA; Columbia University, New York, New NY, USA; New York State Psychiatric Institute, New York, NY, USA; Columbia University Global Health Research Center of Central Asia, Almaty, Kazakhstan; Columbia University Global Health Research Center of Central Asia, Almaty, Kazakhstan; Columbia University Global Health Research Center of Central Asia, Almaty, Kazakhstan; Social Intervention Group, Columbia University School of Social Work, New York, NY, USA; Social Intervention Group, Columbia University School of Social Work, New York, NY, USA

**Keywords:** transgender, nonbinary, HIV, STI, sexual health, trans health, Kazakhstan, Central Asia

## Abstract

**Introduction:**

Trans and gender expansive (TGE) individuals around the world are at increased risk for contracting HIV and sexually transmitted infections (STIs), yet the combination of stigma, accessibility challenges, and a lack of trans-specific, trans-affirming interventions perpetuates rates of infection. Due to the severe paucity of data on TGE communities and HIV in Central Asia, this study describes HIV infections (both known and newly detected) and STIs among TGE in a multicity Kazakhstan study.

**Methods:**

This study utilized behavioral and biological assay data collected in a NIDA-funded clinical trial of a behavioral HIV preventive intervention for substance using cis and trans gay and bisexual men who have sex with men (GBMSM) across three Kazakhstan cities (Almaty, Astana, and Shymkent). We specifically focus on HIV infection, as well as three other STIs (chlamydia, gonorrhea, and syphilis), among 68 TGE individuals who participated in the trial from August 2018 to March 2022.

**Results:**

Findings reveal that while the majority (69%) of TGE participants have undergone HIV testing in their lifetime—with 32% having completed an HIV test in the prior 6 months—over a third (37%) of participants did not know their current HIV status. Fourteen (21%) of the participants were confirmed to be living with HIV, and 11 (79%) of these confirmed infections were reportedly unknown prior to testing. STI testing revealed that 47% of the TGE sample tested positive for chlamydia, gonorrhea, or syphilis, with almost 10% testing positive for more than one of these STIs.

**Conclusions:**

Findings from this study demonstrate high rates of HIV and STIs among TGE individuals in this sample population in Kazakhstan, as well as a discrepancy between HIV status awareness and confirmed HIV diagnosis (with higher rates of confirmed HIV diagnosis). Additionally, the HIV testing rates fall short of the 90-90-90 and 95-95-95 UNAIDS targets for 2020 and 2030, respectively. These results underscore the need for additional research, interventions, and services to address HIV and other STIs and increase testing—concomitantly redressing the conditions leading to marginalization—among TGE in Kazakhstan.

**Clinical Trial Number:**

NCT02786615

## Introduction

The Joint United Nations Programme on HIV/AIDS (UNAIDS) reported that, between 2021 and 2022, the number of people living with HIV had risen globally by 1.3 million, reaching 39 million total [1]. As the rise in HIV rates persists around the world, intervention research must continue to identify and target geographic locations and communities most vulnerable to infection. Compared to the global median HIV incidence of 0.7%, the median prevalence is 7.5% among men who have sex with men (MSM) and 10.3% among TGE people [1]. A 2021 systematic review revealed that up to 25.1% of MSM in Central Asia are living with HIV, but no findings specific to TGE communities were reported [2]. At the time of the study, Kazakhstan, specifically, had experienced the highest increase in HIV infections in Central Asia since 2010 (73% increase from 2010 to 2020), and the prevalence of MSM in Kazakhstan experienced an upward trend from 0.3% in 2009 to 6.2% in 2017 [3, 4].

While research involving MSM in Central Asia and Kazakhstan is beginning to increase, research involving TGE populations in Central Asia remains nascent. A literature review at the time of this writing found only conceptual or formative qualitative work. Wilkinson and Kirey provide a comprehensive examination of the sociopolitical implications of the ‘LGBT’ acronym, committing a specific focus to trans youth in Kyrgyzstan [5]. Additionally, Kirey-Sitnikova’s 2023 qualitative study on trans experience reveals that TGE individuals face extreme hate and stigma across systemic, community, familial, and interpersonal dimensions [6].

While we have examined factors impacting HIV testing rates among MSM and TGE, such as stigma and community connectedness; victimization, discrimination, and disruptions to HIV testing and care; and the intersection of polydrug use and sexual risk behaviors with HIV testing, the findings are shaped more heavily by the experiences of MSM than those of TGE individuals [7, 8]. Additionally, trans and nonbinary people have been found to be significantly more likely to report gender or sexuality-related victimization, discrimination, or both than cis respondents are—these experiences are associated with disruptions in HIV care [8]. There is a dearth of studies and interventions responding to the specific circumstances and situations experienced by TGE communities. Addressing this critical gap and developing an appropriate response warrant attention to key descriptive information—such as the distribution of HIV infection and testing within TGE community.

The data in this study are derived from a stepped-wedge cluster randomized trial of a peer-actuated HIV care intervention for cis gay, bisexual, and other men who have sex with men (GBMSM), and trans, nonbinary, and genderfluid (or otherwise not cis)(TGE) individuals who have sex with men [3, 1, 9]. We conducted a secondary analysis of data from a parent study to specifically focus on the TGE subsample. By examining HIV and STI rates, this paper represents a first step toward establishing the level of need for HIV prevention and care and other sexual health care services among TGE communities in Kazakhstan.

## Methods

### Study Design and Sample

This is a secondary analysis of data obtained during a NIDA-funded clinical trial of a social network-based behavioral preventive intervention for GBMSM individuals at elevated risk for HIV in Kazakhstan (ClinicalTrials.gov: NCT02786615). The parent study sought to enroll a sample of GBMSM individuals in the Kazakhstan cities of Almaty, Astana, and Shymkent. The procedures for sampling, recruitment, and obtaining informed consent are described in detail in prior publications [7, 9]. The eligibility criteria for participation in the trial included (1) being assigned male at birth or identifying as a man at any point in life, (2) residing in one of the three study cities, (3) being 18 years of age or older, (4) reporting one or more incidents of consensual sex with a man in the past 12 months, and (5) reporting one or more incidents of binge drinking, illicit drug use, or both in the past 90 days [10]. Between August 2018 and March 2022, 629 participants who met these eligibility criteria completed a structured interview and 557 completed biological testing for HIV, syphilis, gonorrhea, and chlamydia.

The current analyses focus on the cases for which respondents (1) completed the HIV assay and (2) reported a current gender identity that does not match their sex assigned at birth (i.e., TGE individuals). Aside from current gender identity, there were no significant sociodemographic differences in any of the reported measures between this sample of TGE individuals and the remaining sample in the parent study who identify as cis MSM and completed the study’s HIV testing protocol (*n* = 557). The final sample size for this analysis was 68. All protocols and materials used in the trial were reviewed and approved by the Institutional Review Boards of Columbia University and Al-Farabi Kazakh National University School of Public Health.

### Measures and Study Variables

#### HIV testing history

HIV testing history was assessed using items adapted from the HIV/HCV Testing Domains Measure questionnaire [11]. Participants were first asked whether they had ever received an HIV test (yes, no). If reporting any prior test, participants were asked about the length of time since their most recent test was performed sand were specifically instructed to consider HIV testing other than that provided by the study. Responses were then coded for ever having received an HIV test and having received an HIV test in the past 6 months.

#### HIV and STI statuses

HIV status was assessed through self-reports and biological assays. Participants self-reported their current HIV status as negative, positive, or unknown. For each biological assay (for HIV and STIs), staff provided pretest counseling, testing, and posttest counseling. For HIV testing, trained clinical staff administered an oral rapid test using OraQuick ADVANCE [12]. Additionally, confirmatory testing using blood Western blot (a second-step diagnostic tool) was performed by the participant’s city-run ‘AIDS Center’ and was provided to all participants receiving reactive rapid test results. When testing for gonorrhea and chlamydia, staff collected urine and rectal samples to be tested by local laboratories in each study city using AmpliSens (a nucleic acid amplification test with 99.9% sensitivity and specificity) [13]. To test for syphilis, staff conducted a rapid blood test using Alere Determine Syphilis TP with a capillary blood sample collected via a finger prick [14]. Reactive rapid tests were followed by confirmatory testing in Dermato-Venereological Dispensaries using a nontreponemal test (test for Venereal Diseases Research Laboratory [VDRL] or, in VDRL absence, a Wasserman reaction) and a treponemal test (*Treponema pallidum hemagglutination assay* particle agglutination [TPHA]). All the selected laboratories were certified by the Kazakhstan Ministry of Health for STI testing. For any detected and confirmed HIV infections or STIs, the study provided supportive referral for treatment (e.g., assisted with making appointments for medical care, offered accompanying participants for treatment appointments, etc.).

#### Sociodemographic and background characteristics

Participants self-reported their current residence (Almaty, Astana, Shymkent), gender identity (female/woman, male/man, other, followed by response to ‘please specify’), age (in years), preferred language of survey completion (Russian, Kazakh), legal marital status (single/never married, married, no longer with spouse, other), education completion status (less than high school, high school to some college, baccalaureate or higher degree), current employment status (working full-time, working part-time, student, unemployed), and monthly income (in Kazakh Tenge). Additionally, participants self-reported their lifetime and recent (e.g., past 90 days) incidents of binge drinking, illicit use of drugs, or both [8, 15].

### Statistical Analyses

All statistical analyses were conducted using SPSS Version 28.0 [16]. One way ANOVA or Fisher-Freeman-Halton Exact Tests (due to small cell sizes) were used to assess the significance of differences in participant sociodemographics, background characteristics, and HIV and STI indicators by city of residence.

## Results

The sociodemographic and background characteristics of this sample of 68 TGE individuals in Kazakhstan are summarized in Table 1. As noted in the methods section, this sample of TGE people did not significantly differ from the sample of cis men in the parent study with respect to any of these variables (except for gender identity). Among those who responded outside of the ‘female/woman’ or ‘male/man’ binary gender identities (i.e., ‘Other’ in Table 1), the most common (*n* = 13) were nonbinary identifications (or a corresponding description such as ‘between woman and man’), followed by genderfluid identifications (or an experience corresponding to genderfluidity or genderqueerness such as ‘sometimes a woman and sometimes a man’) (*n* = 11). The remaining participants in this subset were agender (*n* = 2) or remained uncategorized (e.g., ‘don’t know’ or ‘undecided’). Between cities, significant differences in gender identity and language preference were observed in the composition of TGE participants. Among those identified as ‘female/woman’ (*n* = 19), Shymkent had the highest proportion, and Almaty had the highest proportion of those identified outside of the ‘male/man’ or ‘female/woman’ binary (*n* = 17). Finally, among the study cities, Shymkent had the highest proportion of TGE respondents who reported a preference for communicating in Kazakh.

Table 2 presents a summary of HIV testing history, HIV status (both self-reported and confirmed by biological assay), and STI status among this sample of TGE individuals in Kazakhstan. While the majority (69%) have undergone HIV testing in their lifetime and about a third (32%) recently completed an HIV test, these rates indicate that this sample of TGE individuals fell short of the 90-90-90 goals at the start of the study, as well as the current 95-95-95 UNAIDS targets [17]. This is underscored by more than a third (37%) of participants who did not know their current HIV status and 21% of those who tested positive on the biological assay, far exceeding the 4.4% of participants who reported living with HIV. In fact, only three of the 14 (21%) individuals living with HIV reported knowing they were living with HIV.

Table 3 presents a more detailed breakdown of the comparison between self-reported and biologically confirmed HIV status. Of the seven participants who initially reported being HIV-negative but subsequently tested positive, five currently identified as female, and two had ‘other’ gender identities (one person was agender and the other was non-binary). Of the four individuals who were initially unaware of their HIV status and subsequently tested positive, two currently identified as female, and two had ‘other’ gender identities (one of whom was genderfluid and the other of whom was nonbinary). All three respondents who accurately reported living with HIV were currently identified as females.

The sexual health needs of this sample of TGE individuals in Kazakhstan extend beyond HIV, with almost half (47%) of the individuals who completed STI testing receiving positive results for chlamydia, gonorrhea, or syphilis (Table 2 presents a breakdown of STI prevalence by city). Almost 10% (*n* = 6) of this sample tested positive for more than one of these STIs.

## Discussion

To our knowledge, this is the first study in the published research literature focused exclusively on TGE in Kazakhstan that involved participants from multiple cities across the country. Shymkent had the largest proportion of transfeminine participants, as well as higher rates of Kazakh language preference, while Almaty had the greatest proportion of nonbinary participants (although city differences should be interpreted cautiously considering our sample size). We found that 14 out of 68 (21%) TGE individuals in the entire sample tested positive—via rapid and confirmatory testing—for HIV, and of those 14 confirmed positive results, 11 participants (79%) appeared unaware that they were living with HIV (seven said they were negative and the other four did not know their HIV status). The concern for the sexual health of TGE individuals is further amplified given that almost half (47%) of the sample tested positive for chlamydia, gonorrhea, syphilis, or a combination of these infections.

### Limitations

The sample in this study is not generalizable to larger TGE populations in Kazakhstan for several reasons. Participants may have withheld trans or nonbinary identities given local geopolitical trends increasing safety risks and decreasing healthcare access for TGE communities [18]. This study targeted GBMSM individuals involved in illicit use of substances. It is also unclear whether exclusive recruitment using trans-specific and trans-affirming approaches would have reached a different sample of TGE individuals. All GBMSM individuals in this sample were either male at birth or identified as ‘man/male’ at some point in their lives; therefore, this parent study’s emphasis on GBMSM rather than TGE individuals may have failed to actively engage or even excluded several TGE subpopulations (e.g., transfeminine people, nonbinary people, and transmasculine people who do not have sex with men, etc.). These data do not include TGE individuals living in other (especially more rural) regions of the country. Although Almaty, Astana, and Shymkent are populous metropolitan cities in Kazakhstan, and therefore may have larger populations of TGE people, those in more rural or remote settings may have relatively less access to sexual health testing and intervention. Despite, to our knowledge, this study having one of the largest sample sizes of TGE in Kazakhstan to date, the sample size of 68 was still too small, and prohibited any meaningful multivariable analyses. As is true with any self-reporting methodology, participants could have been hesitant to disclose known HIV status (due to stigma, safety, healthcare access, etc.), therefore skewing our discrepant findings between known (self-reported) status and confirmed, tested status.

## Conclusions

Being TGE does not intrinsically drive a greater likelihood of acquiring and transmitting communicable diseases. Rather, the social and developmental conditions faced by TGE lead to the disproportionate burden of HIV and other STIs. Due to the highly marginalized nature of the TGE population, as well as the low representation of TGE voices and experiences in research, it is essential that these findings be expanded upon through intentionally trans-affirming approaches. Even in the face of these limitations, the results from the review of TGE-specific data from this social network-based prevention intervention study yield stark results that call for immediate and ongoing care and attention. This study indicates that TGE people in Kazakhstan will fall well short of the UNAIDS 95-95-95 [17]. The variability in gender identities across cities reinforces that the TGE population should not be treated as a homogenous monolith of individuals; rather, there is heterogeneity in identities, experiences, and prevalence of different types of TGE stigma and discrimination. To continue building health services and systems that provide the care TGE communities deserve, researchers and healthcare workers alike must address this disproportionality by actively prioritizing trans health. As the common trans rallying cry calls, ‘Trans Healthcare is Healthcare’, this study demonstrates the need for research to see trans health as public health. Future research must explore the extent to which increased trans-affirming HIV and STI testing and other sexual health initiatives, outreach, messaging, and structural interventions play a role in decreasing the prevalence of infection among TGE individuals.

## Figures and Tables

**Table 1:** Sociodemographic and background characteristics by Kazakhstan study city (*N* = 68)

	Total sample (*N* = 68)	Almaty (*n* = 28)	Astana (*n* = 14)	Shymkent (*n* = 26)	*p* value for difference among cities

**Gender identity** *n* (%)	37 (54%)	10 (36%)	8 (57%)	19 (73%)	.03
Female/woman	2 (2.9%)	1 (3.6%)	0 (0%)	1 (3.8%)	
Male/man	29 (43%)	17 (61%)	6 (43%)	6 (23%)	
Other					

**Age (yrs.)** *x̅* (*SD*)	29.20 (7.7)	29.1 (7.7)	25.5 (3.7)	31.2 (8.7)	.08

**Preferred language** *n* (%)	57 (84%)	27 (96%)	13 (93%)	17 (65%)	.007
Russian	11 (16%)	1 (4%)	1 (7.1%)	9 (35%)	
Kazakh					

**Marital status** *n* (%)	54 (80%)	25 (89%)	12 (86%)	17 (65%)	.11
Single, never married	6 (8.8%)	0 (0%)	1 (7.1%)	5 (19%)	
Married	7 (10%)	2 (7.1%)	1 (7.1%)	4 (15%)	
No longer with spouse	1 (1.5%)	1 (3.6%)	0 (0%)	0 (0.0%)	
Other					

**Education**^a^ *n* (%)	5 (7.5%)	1 (3 6%)	1 (7.7%)	3 (12%)	.60
Less than high school	32 (48%)	12 (43%)	6 (46%)	14 (54%)	
High school to some college	30 (45%)	15 (54%)	6 (46%)	9 (35%)	
Baccalaureate or higher degree					

**Employment**^a^ *n* (%)	37 (55%)	15 (56%)	9 (64%)	13 (50%)	.61
Working full-time	17 (25%)	6 (22%)	3 (21%)	8 (31%)	
Working part-time	4 (6.0%)	3 (11%)	1 (7.1%)	0 (0%)	
Student	9 (13%)	3 (11%)	1 (7.1%)	5 (19%)	
Unemployed					

**Monthly income (KZT × 1000)** *x̅*(SD)	151 (193)	136 (82.6)	145 (74.3)	169 (299)	.82

**Substance use** *n* (%)	62 (91%)	25 (89%)	13 (93%)	24 (93%)	.99
Binge drinking, ever	56 (82%)	24 (86%)	9 (64%)	23 (89%)	.19
Binge drinking, past 90 days	49 (72%)	20 (71%)	13 (93%)	16 (62%)	.11
Illicit use of drugs, ever	33 (49%)	12 (43%)	9 (64%)	12 (49%)	.45
Illicit use of drugs, past 90 days					

a *N* = 67 (1 missing observation due to ‘refuse to answer’)

**Table 2:** HIV and STI rates by Kazakhstan study city (*N=* 68)

	Total sample (*N* = 68)	Almaty (*n* = 28)	Astana (*n* = 14)	Shymkent (*n* = 26)	*p* value for difference among cities

**HIV Testing** *n* (%)	47 (69%)	20 (71%)	14 (100%)	13 (50%)	.002
Ever Past 6 mos.	22 (32%)	9 (32%)	9 (64%)	4 (15%)	.009

**HIV Status Self-Reported** *n* (%)	40 (59%)	20 (71%)	13 (93%)	7 (27%)	<.001
Negative	25 (37%)	8 (29%)	0 (0%)	17 (65%)	
Unknown/never tested	3 (4.4%)	0 (0%)	1 (7.1%)	2 (8.0%)	
Positive					

**HIV Status Biologically Confirmed** *n* (%)	54 (79%)	24 (86%)	10 (71%)	20 (77%)	.50
Negative	14 (21%)	4 (14%)	4 (29%)	6 (23%)	
Positive					

**Sexually transmitted infection (STI)** ^a^ *n* (%)	14 (23%)	5 (21%)	4 (29%)	5 (22%)	.86
Chlamydia ^a^	5 (8.2%)	1 (4.2%)	1 (7.1%)	3 (13%)	.73
Gonorrhea ^a^	17 (25%)	6 (21%)	5 (36%)	6 (23%)	.63
Syphilis ^a^	*29 (47%)*	*10 (40%)*	*8 (57%)*	*11 (48%)*	*.58*
*Any of the above STIs* ^a^					

a *N* = 61 (7 missing observations due to refusal [ *n* = 1] or sample unable to be assayed/inconclusive [ *n* = 6])

**Table 3:** Self-reported vs. biologically confirmed HIV status (*N* = 68)

		Biologically confirmed	
		Negative (column percentage)	Positive (column percentage)
**Self-reported**	Negative	33 (61%)	7 (50%)
	Unknown/never tested	21 (39%)	4 (29%)
	Positive	0 (0%)	3 (21%)

## Data Availability

Deidentified data used in this study could be made available from EW upon reasonable request.

## Abbreviations

AIDS: Acquired Immunodeficiency Syndrome
HCV: Hepatitis C Virus
HIV: Human Immunodeficiency Virus
LGBT: Lesbian, Gay, Bisexual, Transgender
MSM: Men who have Sex with Men
NIDA: National Institute on Drug Abuse
SGE: Sexual and Gender Expansive
STI: Sexually Transmitted Infection
TGE: Trans and Gender Expansive
TP: Treponema Pallidum
TPHA: Treponema Pallidum Hemagglutination Assay
UNAIDS: United Nations Programme on HIV/AIDS
VDRL: Venereal Diseases Research Laboratory
